# A situation analysis on postmenopausal women’s self-care needs and priorities in Tehran: a population-based study

**DOI:** 10.1186/s12889-023-15040-z

**Published:** 2023-01-14

**Authors:** Masoumeh Simbar, Soheila Nazarpour, Nahid KhodaKarami, Zeinab Nasiri, Farzaneh Rashidi Fakari, Zahra Kiani, Sepideh Keyvanfar, Hamid Alavi Majd

**Affiliations:** 1 Midwifery and Reproductive Health Research Center, Shahid Beheshti University of Medical Sciences, Tehran, Iran; 2 Department of Midwifery and Reproductive Health, School of Nursing and Midwifery, Shahid Beheshti University of Medical Sciences, Tehran, Iran; 3 Department of Midwifery, Chalous Branch, Islamic Azad University, Chalous, Iran; 4 Men’s Health and Reproductive Health Research Center, Shahid Beheshti University of Medical Sciences, Tehran, Iran; 5 General Directorate of Health, The Deputy of Social and Cultural Affairs of Tehran Municipality, Tehran, Iran; 6 Department of Midwifery, School of Medicine, North Khorasan University of Medical Sciences, Bojnurd, Iran; 7 Department of Biostatistics, School of Paramedicine, Shahid Beheshti University of Medical Sciences, Tehran, Iran

**Keywords:** Sexual-reproductive health, Situation analysis, Self-care, Menopause, Women’s health

## Abstract

**Background:**

Women need special care during and after menopause. Due to the emphasis of the World Health Organization on promoting self-care in postmenopausal women, this study aims to analyze the situation and prioritize the self-care needs of postmenopausal women in the Tehran-Iran.

**Methods:**

This was a descriptive-analytical study on 486 postmenopausal women aged 46–85 years living in Tehran in 2021. The Subjects were recruited using a multi-stage sampling method. Data were collected using a socio-demographic and a valid and reliable questionnaire to assess postmenopausal women’s self-care status with four domains including physical health, psychosocial health, reproductive-sexual health, and screening tests. The data were analyzed by SPSS-24.

**Results:**

The mean age of the participants was 62.58 ± 7.75 years. The mean score of self-care was 44.63 ± 21.64% in the postmenopausal women. The lowest score and highest scores were related to psychosocial health (25.12 ± 28.21%) and periodic tests (50.62 ± 24.40%) respectively. There were significant positive correlations between self-care with women’s education level (r = 0.277; *p* < 0.001), husband’s education level (r = 0.258; *p* < 0.001), as well as monthly income (r = 0.153; *p* = 0.001). There was a negative correlation between self-care with the number of children (r = − 0.215; *p* < 0.001). The level of self-care was higher in employed women (*p* = 0.001) and also, in women whose husbands were employed (*p* = 0.012). Multiple linear regression test showed the level of education of the husband (B = 2.72, *p* = 0.038) and the family size (B = -1.54, *p* = 0.023) are predictors of the self-care of postmenopausal women.

**Conclusion:**

The findings showed more than 55% of challenges in the self-care behaviors of postmenopausal women in Tehran. The most and least challenging self-care behaviors were related to psychosocial health and performing periodic tests. The priorities were in psychosocial health and reproductive-sexual health dimensions. Self-care promotion is necessary, especially in postmenopausal women, who need special care due to various physical, psychological, and social changes.

## Background

Menopause is the transition from the fertile to the infertile stage and is one of the most important events in a woman’s reproductive life cycle. This period of life is associated with several physiological changes that may negatively affect the woman’s quality of life [1]. Hormonal changes due to the onset of menopausal signs and symptoms affect both women’s physical conditions and mental health, quality of life and sexual function, and body image. Hence, it is necessary to promote healthy behaviors and lifestyles to improve women’s health during and after menopause [2–4]. In the World Conference of Primary Health Care in 2018, the importance of enabling people to acquire knowledge and skills, and needed resources for maintaining their health was emphasized [5].

Menopause is a physiological condition that affects more than 500 million women aged 42 to 55 years with an average initiation age of 51 years, annually [6] and it is estimated an increase of postmenopausal women to 1.2 billion by 2030 [7]. Nowadays, most women spend more than a third of their lives in post-menopause [1]. Given the increasing number of older women now, resilient efforts are needed to promote self-care behaviors in these women [8]. Women can improve their quality of life by appropriate self-care measures during the menopausal process [9], and promoting self-care during this period of life is emphasized by the World Health Organization [10].

Self-care intervention to improve individuals’ health is introduced as a significant approach [10]. The World Health Organization defines self-care as “the ability of individuals, families, and communities to promote health, prevent disease, maintain health, and cope with illness and disability with or without the support of a healthcare provider” [11].

Nowadays, health systems also emphasize the importance of self-care education [12]. Self-care as a theory focuses on the practice of activities that individuals initiate and perform on their behalf to maintain life, health, and well-being. Dorothea Orem (self-care requisites and self-care behaviors) and Jean Watson (self-compassion within the human caring theory) were among the pioneers of self-care theory [13, 14]. In Kearney and Weininger’s awareness-based model of self-care, self-awareness of people is emphasized as a priority in the field of self-care [15]. Current theories emphasize the role of self-care in different fields and the role of awareness [16]. All of them show the importance of self-care in promoting health.

Self-care existed before formal health systems and plays an important role in health outcomes. For many people in some cultural and social backgrounds, self-care is an established behavior. Self-care is an important component of an individual’s health even if there is access to the health care system. In addition, interventions that were previously only available through healthcare personnel, are now being accessed in the self-care setting [17]. Individuals can solve their health problems through self-care and by raising awareness and modifying their lifestyle [18] and self-care evaluation is a prerequisite for self-care promotion interventions [19].

Self-care is important in all aspects of health and it is particularly essential for populations who are negatively affected by sex, political, cultural, and power dynamics. This is true of sexual reproductive health and rights, where many people cannot decide on their bodies or decide on sexuality and fertility. A safe relationship between quality self-care and quality health care is crucial for vulnerable people to prevent harm [17].

Self-care interventions have increased in the healthcare sector since the beginning of the primary care program with a greater focus on women’s empowerment, improving the intrinsic capacity of older people, and the role of self-care in the management of chronic diseases [17].

The World Health Organization strives that all people in developing and developed countries to benefit from reproductive health services [11]. Self-care in reproductive health means that women can identify their health needs, access appropriate health services, and effectively manage their health conditions, especially seeking reproductive-sexual health care and services [17]. The most important areas of self-care in reproductive health include women’s health in physical, psychosocial, maternal health, postmenopausal health, family planning, infertility, abortion, sexually transmitted diseases, HIV/AIDS, reproductive cancers, Gynecological diseases, and violence [20].

In many areas of sexual and reproductive health and rights, effective self-care with a safe relationship to health care can improve health significantly. These include self-care interventions to regulate fertility (e.g., pregnancy tests and contraception), sexual health promotion (e.g., counseling and information on sexually transmitted infections, and reducing of menopausal symptoms), activities related to prevention and control of disease (e.g., pre-exposure HIV prevention), and treatment (e.g., self-management of abortion) [17]. Self-care interventions increase choice, accessibility, and affordability, as well as opportunities for individuals to make informed decisions about their health and health care [17].

Self-care is one of the key factors in women’s health during menopause. Women can cope with menopausal problems through self-care activities and improve their quality of life [21]. However, some evidence suggests that they lack adequate self-care during menopause [18, 22].

Postmenopausal women experience many concerns about their health and need special care and training services to improve their health and adapt to menopausal changes [23]. On the other hand, providing these services in the health system is costly. In the last three decades, the World Health Organization has placed great emphasis on promoting self-care, especially women’s self-care in reproductive health, mainly in the postmenopausal period, and encourages countries to design and implement self-care programs, particularly in vulnerable populations and in crises [24].

Iranian studies on postmenopausal women show various challenges in physical, mental, and sexual-reproductive health dimensions which need to be identified and prioritized for planning a self-care promotion program. These studies demonstrate are showing significant associations between the severity of menopausal signs and symptoms [25] with the quality of life [3, 26], especially the mental-health dimension [3], sexual health [2], and some medical conditions [27]. The significant associations between anxiety, depression, stress, and postmenopausal women’s body image [28, 29] and sexual health [2, 30] are also demonstrated. Studies have also shown interventions such as hormone therapy [31], and the use of alternative and complementary medicine [32], can improve postmenopausal health, quality of life, and sexual health [33]. Promoting self-care among women during and post menopause including diet correction, using complementary medicine [34] and physical exercise [35], performing specific pelvic floor exercises [36, 37], and developing life skills [23] can alleviate the annoying signs and symptoms of this period of life. Improving self-care behaviors among postmenopausal women can have positive consequences such as increasing quality of life, improving sexual attitude [38] and sexual function [36], reducing anxiety and stress, and even improving body image [4]. But a situation analysis has not been conducted on Iranian postmenopausal women’s self-care behavior.

To the best of our knowledge, there is no comprehensive study on the self-care behaviors of postmenopausal women in Tehran with different economic and social contexts in its 22 districts. Therefore, this study aims to assess the self-care practices among postmenopausal women and their associated factors and also to identify the determinants of self-care to show the needs and priorities for future self-care promotion interventions in Tehran among postmenopausal women. This situation analysis and the needs assessment helps to plan evidence-based and cost-effective interventions.

## Methods

### Study design

This was a population-based descriptive-analytical study.

The subjects of the study were 486 postmenopausal women in two age groups 45–64 and over 65 years, living in all 22 districts of Tehran in 2021.

The inclusion criteria were: resident of Tehran, more than a year has passed since menopause, no known medical and mental condition, and having an appropriate mental state and communication to fill out the questionnaire. According to reliable references [39], menopause was considered the cessation of menstruation for at least twelve consecutive months. The exclusion criteria were incorrect completion of the questionnaire.

#### Sample size

Sampling was performed in all 22 districts of Tehran through the health houses of the Municipality and using a multi-stage sampling method by an online questionnaire (Google form). The sample size was obtained by using the following formula to calculate the sample size.

$$n\ge \frac{z_{1-\alpha /2}^2\left(1-P\right)}{\varepsilon^2P}$$

The minimum sample size was calculated at 385 samples using the formula of descriptive studies and considering the 50% probability of women’s self-care and the Type I error of 0.05 and the absolute error of 0.5.

#### Sampling method

A multi-stage sampling method was used to recruit the subjects of the study. In the beginning, 22 districts of Tehran musicality were considered as the clusters for sampling. Then, three or four health houses were selected by simple random method and using Excel software.

The sample size for each municipality health house was considered 10 to 15 women using the quota method and based on the population covered by the center. The head of the health house in each neighborhood selected the eligible menopausal women using the convenience method of sampling. Afterward, the link to the online questionnaire was sent to the participants by electronic message or WhatsApp, following a cellphone call to them and explaining the goals and process of the study as well as completing the Google form and also obtaining verbal informed consent. Then electronic written informed consent was also obtained from all participants and completing the forms was only possible after giving the informed consent of the participant. For women who were not familiar with the electronic Google forms, the questionnaire was filled out by the head of the health center through a telephone interview. It should be noted that the sampling of this study was performed during the pandemic crisis of Covid-19.

Instructions for sample recruitment were provided to the heads of the selected municipality health houses. Then an online workshop was conducted by the main researcher to train the sampling method and procedure. Then the head of each health house selects ten to fifteen eligible menopausal women from the residents of the neighborhood. The contact number of the main researcher was also provided to the research colleagues to guide and answer the possible questions of the colleagues during the sampling period.

#### Tools for data collection

This online Google form comprised two questionnaires for data collection, including: (1) a socio-demographic questionnaires and (2) a questionnaire to assess Menopausal Women’s Health Self Care (MSCQ-38)

1. ***The socio-demographic questionnaire:*** The socio-demographic instrument comprised questions about personal, social, economic, and anthropometric characteristics of participants. These questions were district, age, weight, height, education, occupation of women, marital status, employment and education of the spouse if married, adequacy of income, housing status, number of children, family size medical history, and condition.
2. ***Menopausal Women Self-Care Questionnaire (MSCQ-38):*** This questionnaire includes 38 items in four domains of physical health (14 items), psychosocial health (6 items), reproductive-sexual health (12 items), and screening tests including periodic tests (6 terms). The items were scored 1 to 3 to the responses of “No, I did not”, “Yes to some extent or I intend to do” and “Yes I did”, respectively. The score ranges were 14–42 for the domain of physical health, 6–18 for the domain of psychosocial health, 12–36 for the domain of sexual-reproductive health, and 6–18 for the domain of screening tests. The total score was 34–190. The score of each domain and the total score were calculated and then converted to the standardized 0 to 100 score using the following eq. (X-Min Score / Max-Min Score) × 100. The higher the score, the greater self-care, and the lower the score, the poorer self-care.

This questionnaire was developed using a deductive approach and based on a review of the guidelines for women’s health, reproductive health, and self-care which were presented on the site of trustworthy organizations such as *the American College of Obstetricians and Gynecologists (ACOG)* [40]*, Royal College of Obstetricians and Gynecologists* [41]*,* Medline Plus [42], World Health Organization women’s health [43], Centers for Disease Control and Prevention (CDC) [44]. Finally, the items of the primary questionnaire were generated based on the updated Women’s preventive Services Initiatives WPSI “2021 recommendations for well-woman care” [45, 46]. This chart is adapted by the members of the advisory panel support for the WPSI including, ACOG, the American Academy of Family Physicians, and the American College of Physicians (ASP). The items were selected and modified to be appropriate for postmenopausal women and based on the guidelines of the above-mentioned reputable organizations.

To assess the validity and reliability of the questionnaire the method described by Pilot and Beck 2010 was used [47]. For the qualitative face validity assessment, 5 menopausal women were asked about the items’ difficulty, irrelevancy, and ambiguity. Afterward, the impact score of each item was calculated and evaluated by the cut-off point of > 1.5. All items of the questionnaire had a score of more than 1.5 and so were considered important by the participants. Then, the content validity of the questionnaire was assessed by 12 experts in midwifery, public health, reproductive health, and nurses. The content Validity of the questionnaire was assessed by calculating the Content Validity Ratio (CVR) and Content Validity Index (CVI). CVR ranged from 0.83 to 1. The modified content validity index of I-CVI for all items ranged from 0.91 to 1, and the S-CVI / Ave score was 0.99.

The reliability of the questionnaire was evaluated by internal consistency assessment by calculating Cronbach’s alpha coefficient, and also stability assessment by test-retest method and calculating intra cluster coefficient (ICC) for all dimensions and the whole questionnaire. Fifteen postmenopausal women completed the questionnaire at two weeks intervals. The results showed the reliability of questionnaire by the Cronbach’s α = 0.89 and ICC = 0.92 for the whole questionnaire (table 1).

**Table 1:** The calculate internal consistency and stability of the Menopausal Women Self-Care Questionnaire (MSCQ-38)

Dimensions	Items	Cronbach’s alpha	ICC	ICC Interval Confidence
Physical health	14	0.93	0.92	0.88–0.94
Psychosocial health	6	0.88	0.94	0.90–0.98
Sexual Health	12	0.84	0.87	0.84–0.90
Screening tests	6	0.92	0.97	0.92–0.98
Total Coefficient for the whole questionnaire	38	0.89	0.925	0.88–0.98

#### Statistical analysis

After filling out the Google forms by the participants in the Google platform, the data were generated in the Excel software in Google Drive. Then the data in the Excel file was converted to SPSS. Then, the data were analyzed using SPSS 24 and by ANOVA, Sheffe, Pearson, and Spearman correlation coefficient tests, and linear multiple regression analysis. *P* values less than 0.05 were considered statistically significant.

## Results

Four hundred and eighty-six postmenopausal women with an average age of 62.58 ± 7.75 (Mean ± SD) years participated in the study. Considering that an electronic questionnaire (google form) was used for data collection, it was not possible to submit the questionnaire until all the questions were answered, we did not have any missing data and samples. The average age of menopause was 50.88 ± 4.11 years. Socio-demographic characteristics of women are shown in table 2.

**Table 2:** Socio-demographic characteristics of the postmenopausal women who participated in the study, Tehran 2021 (*n* = 486)

Variables		Mean ± SD	Minimum	Maximum
Age (years)		62.58 ± 7.75	46.0	**85.0**
Age of menopause (years)		50.88 ± 4.11	39.0	**62.0**
BMI (Kg/m 2)		28.04 ± 4.53	16.90	**45.91**
Duration of marriage (years)		37.79 ± 11.74	0	**65**
Number of children		3.46 ± 1.67	0	**9.0**
Family size		2.73 ± 1.29	0	**8.0**
		**n(%)**		
Age	46–49	15 (3.1)		
	50–54	57 (11.7)		
	55–59	107 (22.0)		
	60–64	106 (21.8)		
	65–69	109 (22.4)		
	≥70	92 (18.9)		
Marital status	Single	11 (2.3)		
	Married	333 (68.5)		
	Divorced	10 (2.1)		
	Widow	132 (27.2)		
Education	No formal education	78 (16.0)		
	Lower Diploma	179 (36.9)		
	Diploma	145 (29.9)		
	Academic	84 (17.2)		
Education of Husband	Illiterate	64 (13.2)		
	Lower Diploma	130 (26.7)		
	Diploma	109 (22.4)		
	Academic	97 (19.9)		
Occupation	Housewife	400 (82.3)		
	Employed	32 (6.6)		
	Retired	54 (11.1)		
Job of Husband	Unemployed	26 (6.6)		
	Employed	142 (33.7)		
	Retired	214 (56.1)		
Homeownership status	The owner	375 (77.2)		
	Non-owner	81 (16.7)		
	Relatives’ house	30 (6.2)		
The adequacy of the family’s monthly income	Inadequate	253 (52.1)		
	Adequate	221 (45.5)		
	More than adequacy/savings	12 (2.5)		

The highest score of self-care was reported for screen tests and the lowest score for psycho-social health self-care among postmenopausal women (Table 3).

**Table 3:** The self-care status of postmenopausal women in different domains of health, Tehran 2021 (*n* = 486)

Self-care Domains	Mean	SD
Physical Health	50.62	24.40
Psychosocial Health	25.12	28.21
Sexual-Reproductive Health	36.54	27.08
Periodic tests and exams	66.34	25.19
Total	44.63	21.64

The self-care assessment of postmenopausal women in the physical health domain showed the highest scores for the items of “measuring height and weight” and “eye and vision care” and the lowest score for “sleep disorders “and “safe driving”.

The self-care assessment in the psychosocial health domain indicated the highest scores for “personal and family relationships” and the lowest score for “suicidal thoughts and actions”. The reproductive-sexual health domain showed the highest level of self-care in “breast self-exam” and the lowest score for performing “AIDS test in high-risk individuals”. The self-care assessment of the women in the domain of periodic tests showed the highest level of self-care for the “blood lipids test” and the lowest score for “colonoscopy” (Table 4).

**Table 4:** The self-care status of postmenopausal women in different measures of each domain, Tehran 2021 (*n* = 486)

Self-Care	Mean	SD
**Physical Health**		
Height and weight measurement	2.44	0.75
Eye and vision care	2.44	0.73
Taking vitamins and minerals	2.38	0.73
Oral health and fluoride consumption	2.25	0.79
Nutrition	2.15	0.83
Use of complementary and herbal medicines	2.02	0.84
Physical activity	2.00	0.82
Using sunlight to absorb vitamin D.	1.92	0.80
lifestyle	1.92	0.80
Avoiding smoking, alcohol, tobacco, and drugs	1.81	0.92
Accident prevention	1.78	0.85
Safe use of mobile phones and the Internet	1.69	0.83
sleep disorders	1.68	0.81
Safe driving	1.68	0.84
**Psychosocial Health**		
Individual and family relationships	1.76	0.83
Anxiety and stress	1.57	0.76
Job Satisfaction	1.56	0.81
Depression	1.55	0.79
physical, sexual, and emotional violence	1.32	0.65
Suicide thoughts and attempts	1.25	0.59
**Sexual-Reproductive Health**		
Breast self-examination	2.136	0.85
Signs and symptoms of menopause	2.13	0.87
Mammography	2.014	0.88
Breast examination by health personnel	1.977	0.90
Hormone Therapy	1.87	0.90
Pap smear in the last 3 years	1.870	0.90
urinary incontinence	1.663	0.84
Pelvic floor disorders	1.504	0.77
Screening for sexually transmitted diseases	1.494	0.78
Prevention of sexually transmitted diseases and AIDS	1.436	0.76
Sexual health and function	1.403	0.73
AIDS test	1.276	0.65
**Periodic Screen Tests**		
Performing blood lipid tests in the last 5 years	2.712	0.57
Diabetes screening in the last 3 years	2.632	0.66
Periodic tests to prevent the risk of chronic diseases	2.53	0.69
Performing thyroid screening in the last 5 years	2.467	0.80
Stool test	1.996	0.93
Intestinal imaging (colonoscopy) in the last 10 years	1.621	0.87

**Fig. 1:**
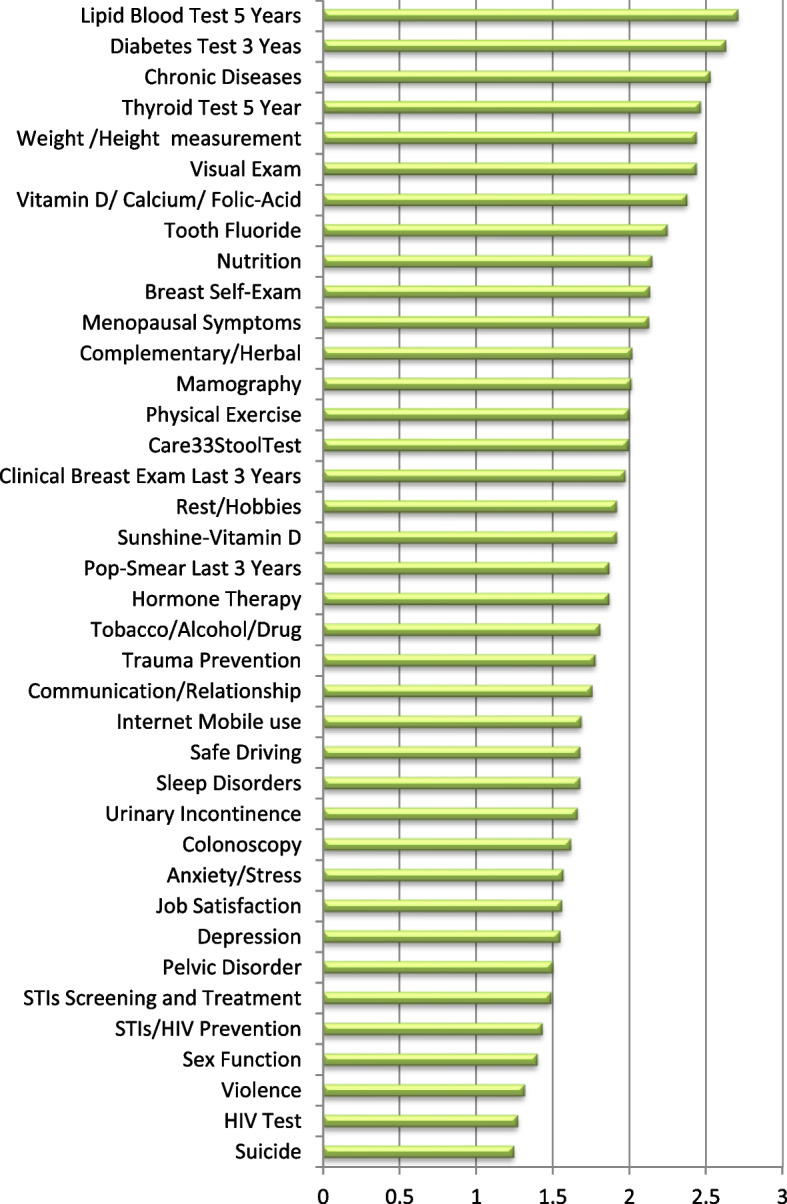
The highest to lowest scores for self-care measures in postmenopausal women Tehran 2021 (*n* = 486)

The self-care assessment of the postmenopausal women in all health domains showed the highest score for the “blood lipids test” and the lowest score for “suicide risk” (Table 4 and Fig. 1).

**Fig. 2:**
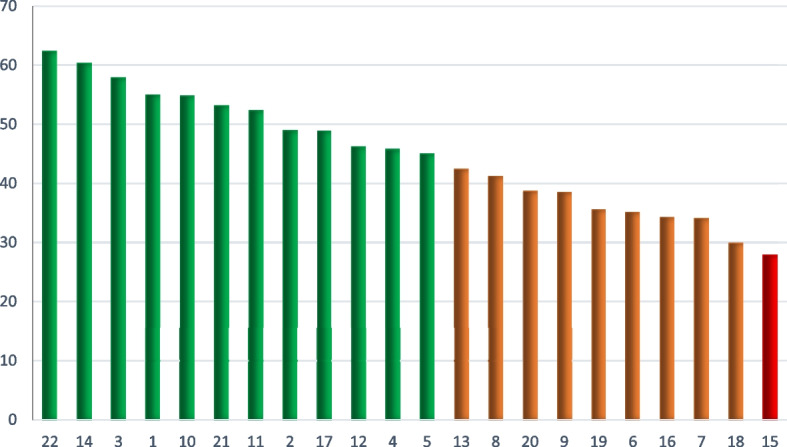
Mean scores of self-care in postmenopausal women in Tehran in 22 districts of Tehran 2021

Analysis of variance showed that the mean scores of self-care were significantly different in districts of Tehran. The highest was found in district 22 and the lowest score was in District 18. Fig. 2 shows the self-care scores in different districts of Tehran from the highest to the lowest in postmenopausal women in Tehran.

Correlation tests showed significant positive correlations between self-care in postmenopausal women living in Tehran with the level of female education (r = 0.277; *p* < 0.001) and the level of husband education (r = 0.258; *p* < 0.001) and their income adequacy. (r = 0.153; *p* = 0.001). There was also a significant negative correlation between the women’s self-care score and the number of children (r = − 0.215; *p* < 0.001) (Table 5).

**Table 5:** Correlations between self-care and some socio-demographic characteristics in postmenopausal women Tehran 2021 (*n* = 486)

Socio-demographic factors	Self-Care	
	*r* ^*p*^*/r*^S*a*^	*p*
	Spearmen Correlation Test	
Age	r ^*p*^ = −0.87	0.056
BMI	r ^*p*^ = −0.050	0.271
Duration of marriage	r ^*p*^ = −0.086	0.081
Age of menopause	r ^*p*^ = − 0.016	0.718
Number of children	r^S^ = − 0.215	< 0.001
Family size	r^S^ = − 0.053	0.247
The adequacy of the family’s monthly income	r^S^ = 0.153	0.001
Homeownership status	r ^*p*^ = − 0.87	0.056
Education	r ^*p*^ = − 0.050	0.271
Education of Husband	r ^*p*^ = − 0.086	0.081

^*a*^*Tests: r* ^p^: Pearson correlation coefficient, *r*^*S*^*:* Spearman’s correlation coefficient

ANOVA and Scheffe post hoc test showed that the self-care of employed postmenopausal women is significantly higher than that of housewife women (*P* = 0.001). Also, in women whose husbands are employed, this score is higher than in women with unemployed husbands (*P* = 0.012) (Table 6).

**Table 6:** Comparison of the self-care scores of postmenopausal women with different socio-economic characteristics in Tehran ((*n* = 486)

Characteristics		n	Mean	SD	ANOVA
Marital status	Single	11	50.12	16.96	ANOVA: df = 3; F = 2.279; *p* = 0.079
	Married	333	46.09	21.77	
	Divorced	10	39.08	27.66	
	Widow	132	40.91	20.83	
Job	Housewife	400	43.08	21.44	ANOVA: df = 2; F = 8.415; *p* < 0.001 Scheffe; *P* = 0.001
	Employed	32	58.47	18.62	
	Retired	54	47.85	21.80	
Homeownership status	The owner	375	44.32	21.79	ANOVA: df = 2; F = 0.208; *p* = 0.813
	Non-owner	81	45.29	20.29	
	Relatives’ house	30	46.67	23.78	
Husband’s Job	Unemployed	26	34.56	20.57	ANOVA: df = 2; F = 4.510; *p* = 0.012 Scheffe; *P* = 0.012
	Employed	142	48.08	21.65	
	Retired	216	45.18	21.08	

The regression test disclosed that only the level of education of the husband (*p* = 0.037) and the family size (*p* = 0.025) predict the self-care of postmenopausal women (Table 7).

**Table 7:** Linear regression test of possible predicting factors of the self-care in postmenopausal women in Tehran 2021 (*n* = 486)

Variables	B	Std. Error	t	*P*
Age	−0.038	0.159	−0.238	0.812
Age of menopause	−0.135	0.203	− 0.664	0.507
Duration of marriage	0.051	0.115	0.446	0.656
Family size	−1.542	0.678	−2.275	**0.023**
The adequacy of the family monthly income	1.046	1.562	0.669	0.504
Education of Husband	2.720	1.304	2.086	**0.038**
Homeownership status	2.042	1.516	1.347	0.179
Husband Job	0.694	1.428	0.486	0.627
Woman Job	−0.776	1.427	−0.544	0.587
Education	2.269	1.399	1.621	0.106

## Discussion

This was the first study to assess the self-care status of postmenopausal women in Tehran. We found the highest self-care in performing periodic screening tests and the lowest self-care in psycho-social health among postmenopausal women. Also, the self-care of postmenopausal women was correlated with the education level of the woman and the husband, and the family’s monthly income. Besides, the family size and the level of education of the husbands were the potential predicting factors for menopausal women’s self-care.

The results showed the average self-care score of 44.63 ± 21.64% in the postmenopausal women in Tehran. This means that postmenopausal women in Tehran meet nearly half of the self-care measures recommended by reliable sources for self-care. To promote self-care among postmenopausal women, using health promotion principles such as women’s empowerment through their health literacy improvement with an emphasis on sexual-reproductive health, women’s engagement from planning to implementation and evaluation, inter-sectorial cooperation, reorientation of the health especially sexual-reproductive in a supportive environment are necessary for self-care promotion in Tehran [48]. Self-care is promoted in many different cities around the world [49, 50] and the psychosocial models for health behavior such as self-care are suggested to be used to assure more successful interventional programs [51].

The results demonstrated psychosocial health self-care is the first priority among the self-care behavior of postmenopausal women. Although menopause is a biological phenomenon, it is also influenced by socio-cultural factors that can cause different experiences and concerns in postmenopausal women from different ethnicities. Signs and symptoms of menopause can lead to negative attitudes towards menopause, and then depression and anxiety [26, 52]. Simbar and colleagues revealed that negative attitudes toward menopause can lead to impaired body image which in turn can predict the onset of depression and anxiety in postmenopausal women [4]. Although the rate of depression, stress, anxiety, violence, suicide, and job dissatisfaction among women is high worldwide, it seems psychosocial self-care is a challenge in Tehran because of two reasons: 1) seeking counseling and Treatment of mental problems is a kind of social stigma, and 2) Psychological counseling is an expensive service [53].

It should be emphasized that screening for mental disorders such as depression, anxiety, stress, and violence is among the recommended self-care behaviors for women by reputable global sources [54]. These mental disorders can be easily screened by standard questionnaires, and inter-sectoral cooperation between health centers and psychological counseling services, and then if necessary, referral to more specialized levels would be possible. Promoting women’s psychosocial health is essential because women are more at risk than men due to biological reasons as well as economic, social, and cultural status and gender stereotypes [55].

Results showed sexual-reproductive self-care as the second priority in postmenopausal women in Tehran. In other words, postmenopausal women meet less than one-third of the recommended criteria for their sexual-reproductive self-care measures. The sexual-reproductive self-care for menopausal women are behaviors related to self-care about menopausal symptoms, hormone therapy, mammography, and screening to prevent breast and cervical cancers, sexually transmitted diseases and HIV, and common postmenopausal conditions such as pelvic floor disorders and sexual dysfunction [56]. The lowest self-care scores were related to performing AIDS testing, sexual function, STIs/HIV prevention, performing STIs/HIV screening tests, and pelvic floor disorders, respectively. It seems performing STIs/HIV screening tests may be low due to the widowhood of some participants and so lack of sexual activity, but misconceptions originating from gender stereotypes and the women’s socio-cultural status seems to be other reasons. For example, there is a misconception among postmenopausal women that urinary incontinence due to pelvic floor disorders is a part of changes during this period of life and it does not require treatment [57], or sexual function should be decreased during the postmenopausal period and counseling for sexual dysfunction or STIs/HIV prevention are not necessary [57, 58]. Even shame and stigma for receiving counseling for sexual dysfunction after menopause can be an important barrier.

It was shown that sexual self-care is a priority and is related to age. A study showed 62.8% sexual dysfunction among post-menopausal women [30]. Contributions of physical, mental, and social factors to sexual dysfunction are shown and reviewed [31, 59]. A phenomenological study on Iranian post-menopausal women indicated 4 concerns about aging, isolation, illness, and inability [26]. The relationship between body image, depression, anxiety, sexual dysfunction, and the aging process among post-menopausal women is well understood [4, 30]. Qualitative studies also showed traditional societies with patriarchal beliefs can intensify concerns and sexual dysfunction [60]. A study on middle-aged Iranian women aged 45 to 65 showed the importance and need for sexual self-care [61]. In addition, despite a common misperception that sex decreases with age, the majority of women aged 65 to 74 have sexual encounters two or three times per month [62] and so this group needs sexual self-care as well. Sexual dysfunction in postmenopausal and elderly women is caused by physical, psychological, and social factors and some medical conditions such as urinary incontinence [23, 27, 31]. Self-care counseling for these women should be planned after examining the conditions and should be based on the factors that cause it.

In addition, the results indicated poor self-care activities such as doing pap smears or breast exams by health care providers or mammograms for cancer prevention among postmenopausal women in Tehran, while breast and cervical cancers are the most common cause of cancer death for women worldwide. In addition, the growing incidence of STIs/HIV as the result of the spread of high-risk sexual behaviors can be controlled by promoting screening tests and exams programs such as periodic breast exams, pap smears, screening and treatment of STIs/HIV, HIV rapid testing and preventing of high-risk sexual health [20, 63].

Findings indicated that the postmenopausal women’s self-care status in the physical health domain was better than other domains and the women pay more attention to nutrition and exercise and vitamin consumption, and weight control, but there were still 44 to 55% gaps in doing self-care behaviors in this domain. Therefore, physical self-care promotion, in some measures such as sleep disorders, accident prevention, avoidance of tobacco/alcohol/ drugs, and the safe use of cell phones that showed the least scores of self-care needs more emphasis. At the same time, these needs can be met by giving information through the media and planning for appropriate education and counseling interventions. Promoting physical health, which is an insensitive area of ​​health, seems to be easily possible through social marketing using mass media [64].

The results showed that more than 66% of the self-care measure in periodic tests and exams were done by the postmenopausal women in Tehran. Although the self-care behaviors in this domain were higher than self-care in other domains, it still faced a challenge of about 34%. To solve this problem, it is possible to provide easy access and affordable laboratory services to women in deprived districts and neighborhoods of Tehran, for instance in public health clinics in the city, and if necessary, follow-up treatment for women in need of further services. There is no doubt that postmenopausal women face not only the complications of this period of life but also aging problems and so they need special geriatric services.

The finding of the present study showed a significant positive correlation between self-care of the postmenopausal women with the education level of the women as well as the education level of their husbands. The regression model also showed the husband’s education as a possible predictor factor for the self-care of postmenopausal women. The findings emphasized the effect of literacy on self-care, which is confirmed in many other studies [65–67]. UNESCO gives emphasizes the importance of education for achieving healthy and a productive life [68]. Therefore, interventions that promote health literacy can improve self-care among people with low levels of education.

The results revealed a significant positive correlation between postmenopausal women’s self-care with monthly family income. Also, the self-care score of employed women was significantly higher than housewives. Besides, the self-care score of women with employed husbands was significantly higher than women with unemployed husbands. Other studies also show the influence of economic and occupational status on individuals’ health and self-care behaviors [65, 67]. Promising economic conditions are often associated with more access to health services such as periodic screening [3].

The finding also demonstrated a significant negative correlation between postmenopausal women’s self-care with the number of children. Also, family size was a potential positive predictor for women’s self-care. It seems more children can increase parental responsibility; financial problems and stress [69]. A study showed more children are associated with lower quality of life, which was attributed to the women’s greater responsibilities in large families. The numerous responsibilities of women in large families can leave them with less time and opportunity for self-care [3].

### Strengths and weaknesses

This study has several noticeable features that make it stand out from other studies. These include addressing the subject of self-care, which is an important focus of the World Health Organization in recent years [11], and also addressing women’s health, which is a requisite of the sustainable development goals [70].

One other strength of this study is the use of a valid and reliable questionnaire that cover all areas of self-care. The strength of this research was the assessment of a large sample that was taken from different districts and neighborhoods of Tehran with the support and coordination of the General Directorate of Health of Tehran Municipality and authorities of Tehran municipal health houses. However, the sample for each district was not large enough to for comparing self-care in different districts or regression analysis for each district.

One of the limitations of this study was the focus on women’s health, which was proposed and implemented in terms of the importance of women’s empowerment in the field of health. While the implementers of this project acknowledge that people with disabilities in the field of reproductive health have special needs in reproductive-sexual health. In this regard, it is suggested that a separate study be conducted on the self-care status of women with disabilities in the future.

Besides, we did not exploratory factor analysis (EFA) or Confirmatory Factor Analysis (CFA) since the self-care dimensions and items are known, and EFA or CFA are compulsory for exploring or confirming the construct of a valid instrument. But EFA or CFA are possible and suggested to be performed in future studies.

The wide age range of women who participated was another limitation of this study. However, the participants were from two age groups postmenopausal women aged 45–64 and more than 65 years old. But the items for self-care were similar and besides in the comparison of these two groups’ self-care behavior they were not significantly different. Perhaps, if the sample had a narrower age range, the results would make sense more.

Another limitation of the project was the use of self-report questionnaires, which may be less accurate than an interview or reviewing the documents. An attempt was made to increase the accuracy of completing the questionnaires by training and sending instructions to sampling colleagues and participating women.

A complication of the study coincided with the Covid-19 pandemic crisis that could affect the change in some health behaviors such as referring to health centers and hospitals for periodic screening tests, examinations, and care.

## Conclusion

Only half of the post-menopausal women in Tehran practice standard postmenopausal self-care. Among those who practice self-care, the majority do periodic tests, and only very few practice psycho-social aspects of self-care. Therefore, it is necessary to pay special attention to self-care in national health policies for promoting public health, especially women’s health. These measures are especially important for postmenopausal women who need special attention due to various physical, psychological, and social changes. In this regard, creating a supportive environment to promote self-care, inter-sectorial cooperation, reorientation of health services, and strengthening community actions to promote self-care, as well as creating skills for postmenopausal women are suggested.

Also, self-care in postmenopausal women living in Tehran was related to some personal and social characteristics such as the education level of women and their husbands, monthly income, employment, and family size. Therefore, targeting vulnerable women from low socio-economic groups for self-care promotion interventional programs is also recommended.

## Abbreviations

STIs/HIV: Sexually Transmitted Infections/ Human Immunodeficiency Virus
MSCQ-38: Menopausal Women Self-Care Questionnaire (38 items)
ACOG: American College of Obstetricians and Gynecologists
CDC: Centers for Disease Control and Prevention
WPSI: Women’s Preventive Services Initiatives
ASP: American College of Physicians
CVR: Content Validity Ratio
CVI: Content Validity Index
S-CVI: Scale Content Validity Index
I-CVI: Item Content Validity Index
ANOVA: Analysis of Variance
SPSS 24: Statistical Package for the Social Sciences (version 24)
